# The effects of vitamin E supplementation on malondialdehyde as a biomarker of oxidative stress in haemodialysis patients: a systematic review and meta-analysis

**DOI:** 10.1186/s12882-021-02328-8

**Published:** 2021-04-09

**Authors:** Peter Bergin, Aoife Leggett, Chris R. Cardwell, Jayne V. Woodside, Ammarin Thakkinstian, Alexander P. Maxwell, Gareth J. McKay

**Affiliations:** 1 Centre for Public Health, Queen’s University Belfast, Belfast, UK; 2 Department of Clinical Epidemiology and Biostatistics, Faculty of Medicine, Ramathibodi Hospital, Mahidol University, Bangkok, Thailand; 3 Centre for Public Health, Queen’s University Belfast, Royal Victoria Hospital, Belfast, BT12 6BA UK

**Keywords:** Haemodialysis, Oxidative stress, Malondialdehyde, Vitamin E, Tocopherol, Biomarker

## Abstract

**Background:**

Haemodialysis (HD) patients tend to have higher levels of oxidative stress (OS), associated with increased morbidity and premature mortality, compared to the general population. Levels of malondialdehyde (MDA), a biomarker of OS, are reduced by the antioxidant properties of vitamin E (VE) but outcomes from randomised control trials of VE supplementation on MDA in HD patients have been inconsistent.

**Methods:**

We undertook a systematic review and meta-analysis of adult HD patients from VE supplementation studies with measures of MDA. The following search criteria of MEDLINE and EMBASE were considered from inception to January 2020: ‘dialysis’ AND ‘Vitamin E OR tocopherol’ AND ‘malondialdehyde OR MDA’. Two reviewers independently extracted study data and assessed risk of bias. Mean MDA levels and standard deviation were determined before and after VE supplementation. Standardised mean difference (SMD) and standard error were calculated as the within person difference and units of measure were not consistently recorded across all studies. The SMD were pooled using random effects meta-analysis.

**Results:**

The SMD of MDA levels from 18 comparisons was significantly lower in HD patients following VE supplementation (− 1.55; confidence interval: − 2.17 to − 0.94, *P* < 0.00001). There were significant levels of heterogeneity between studies (I^2^ value = 91%; P < 0.00001) with evidence of potential publication bias toward smaller studies.

**Conclusions:**

Our findings support the use of VE to reduce the effects of OS in HD patients although high levels of heterogeneity and variation in the methodological approaches used by some studies highlight the need for further investigation.

## Background

Oxidative stress (OS) is defined as “an imbalance between oxidants and antioxidants in favour of the oxidants that leads to a disruption of redox signalling and control and/or molecular damage” [1]. Endogenous oxidants such as reactive oxygen species (ROS), generally manifest from unstable superoxides and hydroxyl free radical by-products of intracellular metabolic reactions. They may also form because of environmental factors, including ultraviolet radiation and nitrogen dioxide air pollution. Small and acute perturbations are generally well tolerated and reversible through redox signalling, a process known as physiological oxidative eustress [2]. Chronic and excessive exposure, known as oxidative distress, can disrupt redox signalling, damage biomolecules and manifest with significant pathological consequences [3]. Patients with end-stage renal disease (ESRD) accumulate uraemic toxins with progressive loss of renal function. Some uraemic toxins, such as homocysteine and advanced glycation end-products (AGEs), are difficult to remove during haemodialysis because significant proportions are protein bound. Uraemic toxin accumulation has been associated with OS that is associated with cardiovascular disease (CVD) progression in ESRD patients [4].

Antioxidants are found exogenously in plant-based whole foods such as fruits, vegetables, or nuts and supplementation is a commonly used intervention for the management of OS in an effort to increase total antioxidant capacity (TAC) beyond that of a normal diet [3, 5]. Vitamin E (VE) is a lipid soluble antioxidant composed of eight compounds of similar structure, four tocopherol and four tocotrienol (TT) derivatives including α-, β-, γ- and δ-tocopherol and α-, β-, γ- and δ-tocotrienol. It is primarily bound to the hydrophobic interior of the cell membrane where it offers protection against injurious membrane oxidation by free radical scavenging [3] through the donation of an electron to the products of lipid peroxidation [6]. Alpha tocopherol (AT) is the most abundant and biologically active form of VE [7], primarily due to its preferential re-secretion in the liver [8]. The majority of VE supplements contain AT only, and the terms AT and VE are often used interchangeably despite the unique properties of the other VE derivatives, such as α-tocotrienol in neuroprotection [9].

Malondialdehyde (MDA) is a secondary by-product of cellular lipid peroxidation of polyunsaturated fatty acids [10] formed within the intracellular space by the degradation of membrane phospholipids [11]. Elevated lipid peroxidation can overwhelm the antioxidant defence system and trigger cell apoptosis and pathological processes [12] leading to elevated serum MDA levels that reflect increased free radical production [13]. Patients with ESRD, including persons managed by chronic haemodialysis (HD), have both higher CVD and all-cause mortality [14]. Increased MDA and other biomarkers of OS contribute to elevated morbidity and mortality in HD patients [15], a process likely to commence at an early stage of renal decline [16] through ischaemic glomerular and tubular injury exacerbated by malnutrition, antioxidant loss during dialysis, bacterial/toxic products in dialysate, and hypersensitivity reactions [17]. Increased OS has also been associated with repeated iron infusions, anaemia, and dialyzer bio-incompatibility [18]. MDA is recognized as a clinically reliable marker of OS associated with the pathology of several conditions including diabetes and CVD [12]. MDA represents a good predictor of survival in HD patients, with lower levels associated with decreased mortality in long-term follow-up [19].

Measurement of MDA levels has largely been based on a reaction with thiobarbituric acid (TBA) resulting in the production of “thiobarbituric acid reactive substances” (TBARS) that can be quantified by colorimetric or fluorimetric excitation. This simple and cost-effective approach, has been used routinely for clinical analysis, despite reported limited sensitivity and specificity that can be confounded by chemically reactive carbonyl group–containing compounds, such as sugars, amino acids, bilirubin, and albumin, which interfere with the colorimetric and fluorimetric MDA measurement [20]. Other biomarkers of lipid peroxidation also include reactive intermediates such as the arachidonic acid derived prostaglandin F2 alpha isomer isoprostanes iron reactive oxygen metabolites (Fe-ROMs) and diacron reactive oxygen metabolites (d-ROMs). F2α isomer isoprostanes are widely used and demonstrate similar efficacy to MDA as markers of OS. Fe-ROMs and D-ROMs directly quantify specific peroxidation products in contrast to MDA and isoprostane methods where end products of OS are measured, however both isoprostane and MDA remain commonly used indicators of OS [20].

Bayés and colleagues demonstrated the potential benefits of VE supplementation in HD patients with malnutrition and lower serum VE levels [21]. The potential benefits of MDA as a biomarker of OS beyond traditional CVD risk factors was also reported in HD patients [22, 23]. However, the results from previous interventional studies have been inconsistent. Lu and colleagues reported no differences in oxidised protein levels between placebo and daily VE consumption (800 IU for 6 months) in HD patients [24]. In contrast, 300 mg of VE taken three times per week for four weeks reduced oxidised protein levels and indicators of DNA damage in HD patients [25]. Furthermore, daily supplementation with 400 IU of VE for 4.5 years showed no effect on cardiovascular outcomes in high-risk patients [26]. Davi et al. demonstrated a reduction in the F2 alpha isomer isoprostane, 8-isoPGF_2_α, following VE supplementation in diabetic patients [27], however data on VE supplementation and lipid peroxidation in HD patients is scarce. As such, the aim of this meta-analysis (MA) was to determine the effects of VE supplementation on MDA levels in HD patients and its potential as a treatment modality to reduce CVD risk and all-cause mortality in HD patients.

## Methods

### Data sources and search criteria

**Fig. 1:**
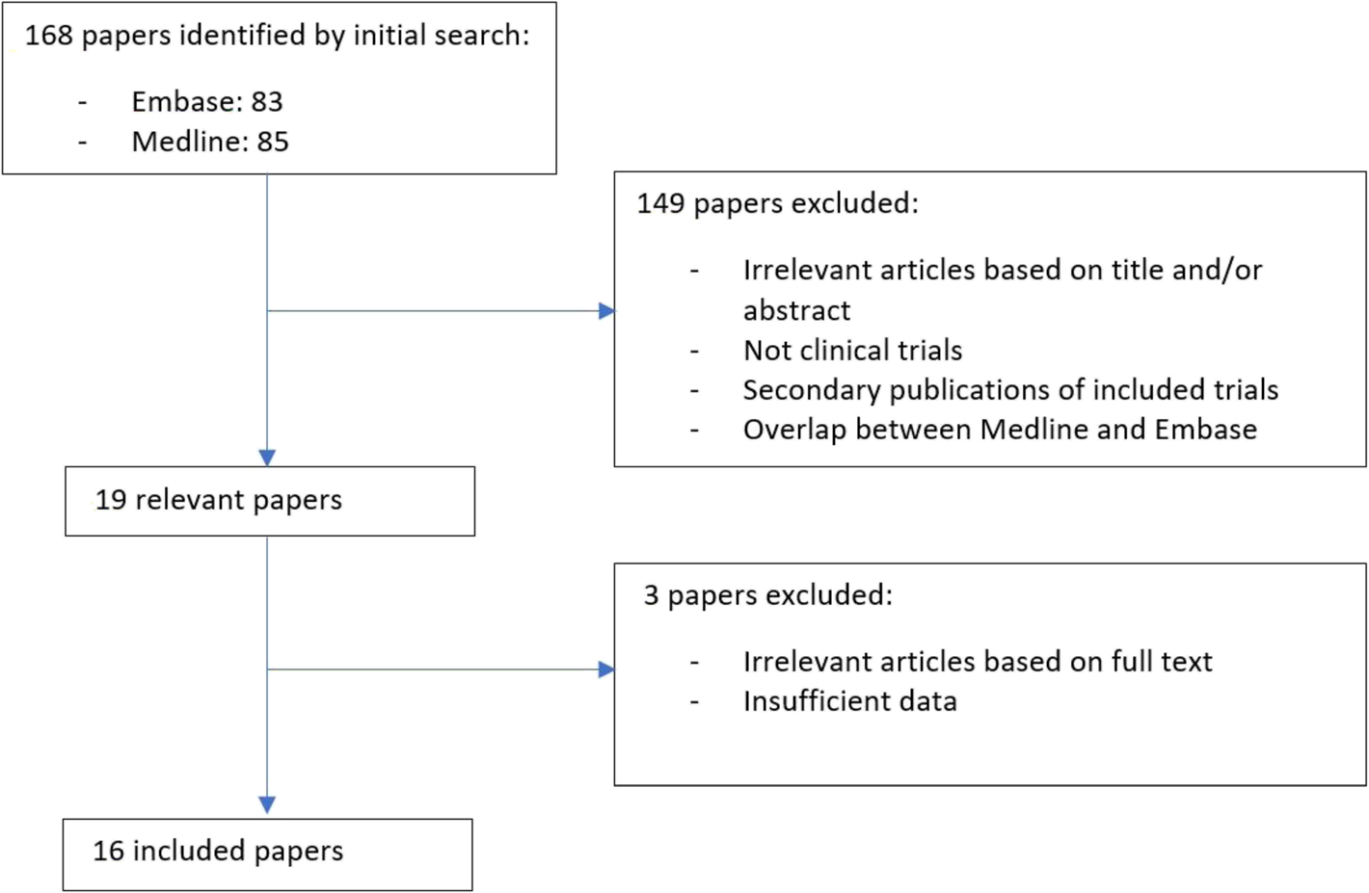
Flow chart of trial selection and inclusion process

PubMed (Medline) and EMBASE were searched from inception to January 2020 by two independent reviewers (AL and PB). Search terms were as follows: ‘dialysis’ AND ‘vitamin E OR tocopherol’ AND ‘malondialdehyde OR MDA’ (Fig. 1). Studies were limited to the English language and human and clinical trials.

### Study selection

Full articles were reviewed if a decision could not be determined from initial screening of titles and abstracts. Disagreement between both reviewers was discussed and resolved by a third party (GM). The list of references from selected articles and previous reviews were also considered to identify additional relevant studies that may have been missed during the initial search.

Comparative studies either randomised controlled trial (RCT), non-RCT, or before-after trials were eligible if they met the following inclusion criteria: studied in adult HD patients, had compared any form of VE supplement with placebo/control regardless of dosage and course of supplementations, and had MDA levels as the outcome of interest. Studies were ineligible if they used VE-coated membrane dialyzers, only investigated dietary or serum VE levels, or did not report the number of participants and their mean MDA levels and standard deviation (SD), following attempts to contact the corresponding author.

### Interventions and outcomes of interests

The form of VE supplementation within included studies was variable and comprised α-tocopherol, α-tocopherol acetate and tocotrienol. Several studies failed to specify the form of VE used and as such, all interventions were considered collectively as VE supplementation. Variation in the substrate used for the measurement of MDA included erythrocytes, platelets and plasma.

### Data extraction

Data extraction included study authors, publication year, country of origin, trial design, form of VE, dose of VE, treatment regimen and duration, mean participant MDA levels ± SD before and after VE supplementation, and number of participants.

### Data synthesis and analysis

In each study, the number, mean MDA levels and SD was determined before and after VE supplementation. The difference in mean and standard error (SE) was calculated treating the before and after group as independent, because the within person difference and SD of the within person difference and the units of measure were not consistently recorded across all studies, and was converted to a standardised mean difference (SMD) and SE. The SMD was pooled using random effects models implemented in RevMan (version 5.3). Heterogeneity between studies was tested using the Chi-squared test for heterogeneity and assessed using the I^2^ test statistic and publication bias using funnel plots. Within person analysis was not performed as paired data was unavailable for all studies. As a result, dependent observations were treated as independent with mean MDA levels compared at baseline and post intervention. For consistency, only individuals from the intervention arm of RCTs were included. Sensitivity analyses were determined a priori to evaluate the robustness of our findings. These included the form of VE supplemented (synthetic vs natural), study design (RCT vs non-RCT), route of administration (oral vs intramuscular (IM)), and duration of VE therapy (≤ 1-month vs > 1 month). Post hoc subgroup analyses also included VE supplementation or co-supplementation, and forms of MDA biomarker (serum/plasma MDA vs non-serum/plasma MDA).

## Results

### Study and participant characteristics

Nineteen studies were shortlisted from the 168 that met the initial search criteria, of which 16 were considered relevant (Fig. 1, Table 1). Two of the 16 studies were RCTs that included two separate treatment arms that facilitated consideration of 18 comparisons in total. One RCT of HD patients consisted of a treatment arm with AT as a monotherapy (Asemi^a^ et al) or AT and omega 3 fatty acids co-supplementation (Asemi^b^ et al) [43]. The second RCT included HD patients with AT monotherapy (Ahmadi^a^ et al) and AT plus alpha lipoic acid (ALA) co-supplementation (Ahmadi^b^ et al) [41]. The total number of participants was 338, ranging from five to 41 for each comparison. Mean therapy duration was two months and ranged from one day to six months. The year of publication spanned thirty-two years from 1984 to 2016. Eight studies were performed in Europe, nine in Asia and one in the United States. Five trials delivered VE IM, 11 delivered VE orally, with two modes of delivery unspecified. The mean VE dose was 384 mg and ranged from 220 mg to 1080 mg. Seven trials delivered VE as co-supplementation and 11 delivered VE as monotherapy. Fourteen of the studies included in our MA used the TBARS assay to measure MDA, while a single study used high performance liquid chromatography (HPLC).

**Table 1:** Characteristics of studies included

Study	n (cases)	Dose	Route	Form	Therapy Duration	Additional Treatment	MDA Mean ± SD Before	MDA Mean ± SD After	Biomarker	Country
Giardini et al., 1984 [28]	19	300 mg/day 330 IU	IM	α-tocopherol acetate	15 days		19.53 ± 7.83 μg/ml	0.46 ± 0.36 μg/ml	Erythrocyte MDA	Italy
Taccone-Gallucci et al., 1985 [29]	9	300 mg/day 330 IU	IM	α-tocopherol acetate	15 days		6.17 ± 1.3 μg/ml packed RBC	0.63 ± 0.2 μg/ml packed RBC	RBC MDA	Italy
Lubrano et al., 1986 [30]	9	300 mg/day 330 IU	IM	α-tocopherol acetate	15 days		6.16 ± 1.29 μg/ml	0.69 ± 0.13 μg/ml	Erythrocyte MDA	Italy
Taccone-Gallucci et al., 1986 [31]	10	300 mg/day 330 IU	IM	α-tocopherol acetate	15 days		16.92 ± 3.36	7.92 ± 5.80 T2 g/mg	PBMC MDA	Italy
Taccone-Gallucci et al., 1989 [32]	10	300 mg/day 330 IU	IM	α-tocopherol acetate	15 days		2.35 ± 1.46	0.5 ± 0.2 T2 μg/mg	Platelet MDA	Italy
Hassan et al., 1995 [33]	6	400 IU daily (268/449 mg)	Oral	unknown	40 days	Vitamin A 4500 IU daily + Vitamin C 750 mg daily	1.67 ± 0.37	0.51 ± 0.1	RBC MDA	Iraq
Yukawa et al., 1995 [34]	5	600 mg/day 900 IU	Oral	α-tocopherol	2 weeks		4.0 ± 0.6 nmol/ml	3.3 ± 0.6 nmol/ml	MDA-LDL	Japan
Cristol et al., 1997 [35]	7	500 mg/day 750 IU	Oral	α-tocopherol	6 months	EPO + Iron	1.7 ± 0.1 nmol/ml	1.2 ± 0.1 nmol/ml	Serum MDA	France
Inal et al., 1999 [36]	36	300 mg/day 330/450 IU	Oral	unknown	3 months	EPO	4.05 ± 0.05 nmol/ml	3.81 ± 0.09 nmol/ml	Plasma MDA	Turkey
Roob et al., 2000 [37]	22	1080 mg 1200 IU	Oral	all-rac-α-tocopherylacetate	One dose	EPO + Iron	1.20 + − 0.28 μmol/L	1.09 ± 0.26 μmol/L	Plasma MDA	Austria
Bayes et al., 2001 [38]	16	400 mg 3/week 440/600 IU	Oral	unknown	3 months	EPO + Iron	5.76 ± 1.83 μmol/L	3.89 ± 1.14 μmol/L	Plasma MDA	Spain
Uzum et al., 2006 [39]	19	300 mg/day 330/450 IU	Oral	unknown	20 weeks		2.77 ± 0.87 nmol/mL	2.2 ± 0.767 nmol/mL	Plasma MDA	Turkey
Youzbaki et al., 2010 [40]	18	400 mg/day 600 IU	Oral	α-tocopherol	3 weeks		0.059 ± 0.027 μmol/L	0.031 ± 0.021 μmol/L	Serum MDA	Iraq
Ahmadi^a^ et al., 2013 [41]	17	400 IU (268/449 mg)	Unknown	unknown	2 months		4.4 ± 1.6 μmol/L	4.7 ± 1.2 μmol/L	Plasma MDA	Iran
Ahmadi^b^ et al., 2013 [41]	24	400 IU (268/449 mg)	Unknown	unknown	2 months	600 mg ALA	4.9 ± 1.6 μmol/L	4.5 ± 1.3 μmol/L	Plasma MDA	Iran
Daud et al., 2013 [42]	41	220 mg/day (180 mg TT + 40 mg AT)	Oral	TT (⍺-TT, β-TT, 훾-TT, 훿-TT) + α-tocopherol	16 weeks		3.01 ± 4.65 μM	2.89 ± 3.65 μM	Plasma MDA	USA
Asemi^a^ et al., 2016 [43]	30	268 mg/day 400 IU	Oral	α-tocopherol	3 months		3.2 ± 1.0 μmol/L	3.7 ± 1.5 μmol/L	Plasma MDA	Iran
Asemi^b^ et al., 2016 [43]	30	268 mg/day 400 IU	Oral	α-tocopherol	3 months	Omega 3 s	3.5 ± 1.2 μmol/L	3.4 ± 1.4 μmol/L	Plasma MDA	Iran

### Clinical outcomes, quality and Bias assessment

**Fig. 2:**
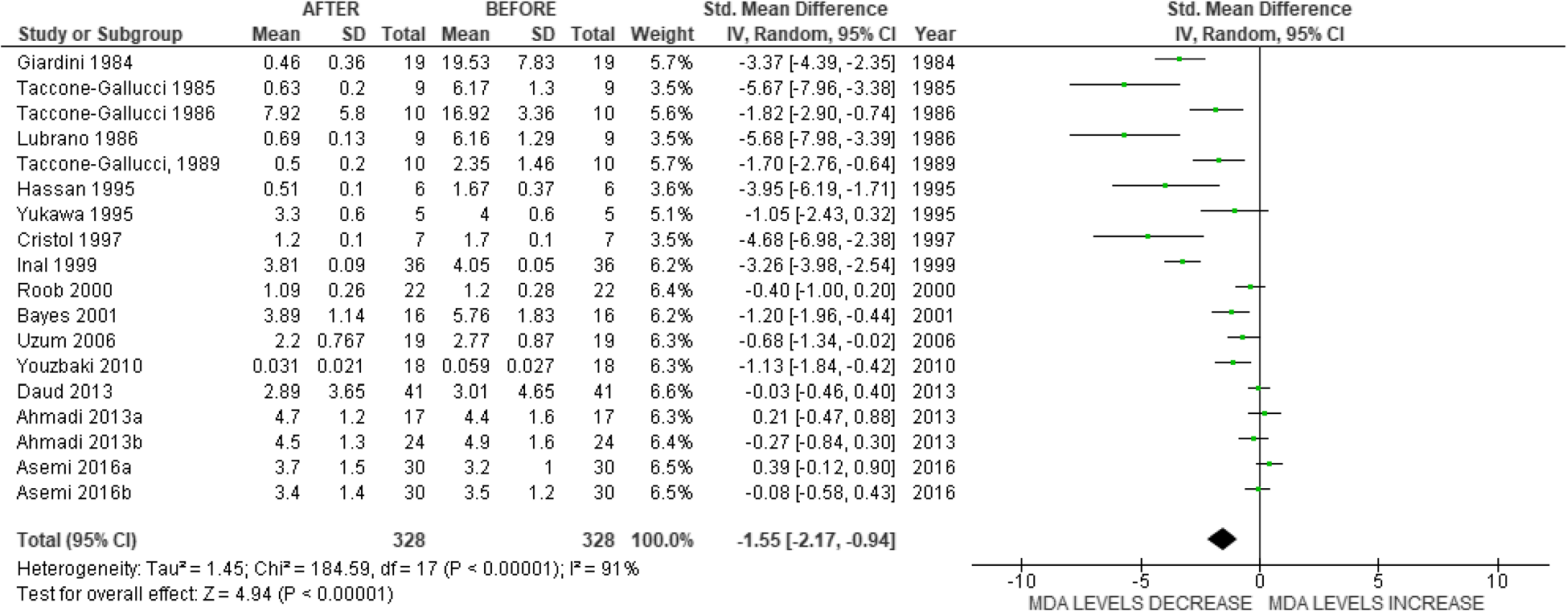
Forest plot for the individual and meta-analyses effect of VE supplementation on MDA levels

**Fig. 3:**
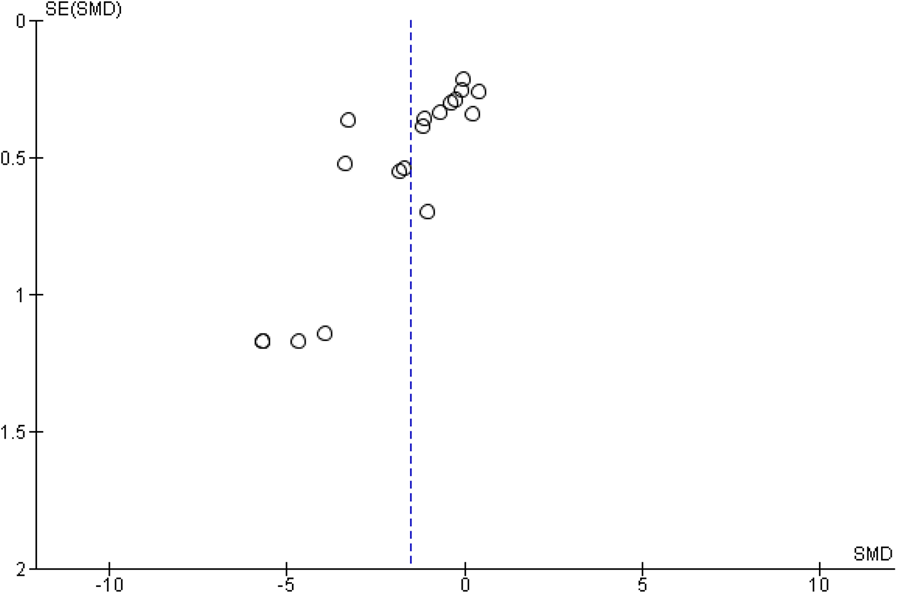
Funnel plot for standardised mean difference of MDA levels for VE supplementation studies

The overall effect estimate resulted in a SMD = − 1.55 (confidence interval (CI) -2.17 to − 0.94, *P* < 0.00001; Fig. 2) with high levels of heterogeneity observed (I^2^ = 91%; P < 0.00001). The funnel plot was considered asymmetrical around the true population effect and there was no narrowing of SMD as SE decreased (Fig. 3). Sensitivity analysis, which included the removal of a single study at a time, identified Inal et al., 1999 [36] to exert the largest effect which resulted in a reduced SMD of − 1.37 (CI − 1.93 to − 0.80), when excluded. Subgroup analyses demonstrated significant negative associations between VE supplementation and MDA levels in oral and IM administration, non-RCTs, serum/plasma and non-serum/plasma MDA biomarkers, study duration ≤1 month and > 1 month, and synthetic form of VE. The negative association was not significant for RCTs studies that administered the natural form of VE (Table 2).

**Table 2:** Subgroup analyses for the effect of VE supplementation on MDA levels

Subgroup analyses	Number of trials	SMD	95% CI	I^2^ value
Route				
IM	5	−3.34	−4.73, − 1.95	81
Oral	11	−1.16	− 1.86, − 0.46	90
MDA biomarker				
Serum/plasma	11	−0.79	−0.40, − 0.19	90
Non-serum/plasma	7	−3.05	−4.21, −1.89	78
Duration				
≤ 1 month	8	−2.29	−3.34, −1.25	87
> 1 month	10	−1.01	−1.75, −0.28	91
Co-supplementation				
Monotherapy	11	−1.52	−2.32, −0.71	90
Combination	7	−1.69	−3.03, −0.35	92
Vitamin E form (if known)				
Synthetic	6	−2.84	−4.33, −1.35	90
Natural	6	−0.58	−1.26, 0.10	82
Study Design				
RCT	4	−0.05	− 0.33, 0.22	33
Non-RCT	12	−2.53	−3.38, −1.67	87

## Discussion

The findings from our MA identified a significant SMD of − 1.55 in MDA levels in HD patients following VE supplementation. Lower levels of OS have been associated with less comorbidities and CVD in HD patients, as OS is known to contribute to atherosclerosis via oxidation of LDL [44]. MDA is a clinically important quantitative measure of OS and an indicator of the efficacy of VE supplementation as a potential intervention in HD patients. However, our findings should be interpreted with caution to understand their limitations and inform best clinical practice.

VE has been reported to exert pro-oxidative effects at high doses [45] with supplementation of 745 IU/day for six weeks leading to increased pro-oxidant ferrous oxidation xylenol (FOX) by 27% [46]. A MA in 2005 of mainly chronic disease populations found VE supplementation ≥400 IU/day for at least one year was associated with increased all-cause mortality [47]. The pro-oxidative effect of VE was proposed as the underlying mechanism due to imbalanced TAC and inhibition of human cytosolic glutathione S-transferases, which contribute to the detoxification process [48]. The VE pro-oxidant hypothesis may also explain the unexpected findings of Asemi^a^ et al., whereby 400 IU/day of AT was associated with increased MDA levels by 0.4 ± 1.5 μmol/L [43]. High dosage VE may be associated with an increased risk of bleeding and clinical events such as haemorrhagic stroke [49] and with HD patients already at an increased risk of bleeding [50], this may be exacerbated further by VE supplementation. Co-supplementation of VE with co-enzyme Q reduced these pro-oxidative effects [45] and may explain why 400 IU of AT daily co-supplemented with 1250 mg of omega-3 fatty acids reduced MDA levels by 0.1 ± 1.2 μmol/L compared to AT monotherapy which saw MDA levels increase by 0.4 ± 0.5 μmol/L [43]. A 2014 MA reported low doses of VE below 400 IU/day when combined with other vitamins and minerals, was associated with reduced all-cause mortality [51]. However, after subgroup analysis, this effect was observed only in healthy people, limiting its generalizability in a HD population. Evaluation of antioxidants in isolation fails to consider the potential synergistic effects of other nutrients and the safety concerns associated with VE supplementation, especially at high doses [52]. Caution should be exercised regarding unfettered supplementation, especially when in excess of the recommended daily allowance (RDA) [53], although HD patients tend to have higher antioxidant requirements than the general population [54].

Subgroup comparisons of co-supplementation and monotherapy identified no significant difference in effects, although co-supplementation had a slightly greater effect than monotherapy alone (Table 2). Three of the co-supplementation studies included erythropoietin (EPO) and iron and a single study included EPO without iron. EPO and iron are used to treat renal anaemia in patients with CKD [55] and the severity of renal anaemia is correlated with lipid peroxidation status. The antioxidant effects of EPO has been reported to ameliorate renal anaemia and OS in HD patients [56]. Its inherent antioxidant properties include cytoprotective effects that stimulate increased red blood cell count [57]. The increase in OS by iron administration in HD patients is well established and the co-administration of iron with EPO may result in reduced antioxidant effects [18]. VE reduces the pre-oxidative action of iron infusion in HD patients [58], while increasing EPO responsiveness and haemoglobin levels [59].

Gamma tocopherol (GT) is estimated to account for approximately 70% of VE in the average American diet, and is found largely in soybean and other vegetable oils [60]. AT is found in foods from arguably healthier sources, such as nuts and spinach and AT supplementation has been shown to lower plasma GT levels that leads to pro-oxidation from TAC imbalance [61]. While GT exhibits unique protective properties [62], the AT-dominant supplementation may rebalance a skewed GT-dominant diet. When compared to each isolated form, the greatest inhibition of oxidised-LDL (ox-LDL) mediated ICAM-1 expression was observed with a mixture of GT and AT suggesting a combination of tocopherols may have a greater anti-oxidising effect than AT alone [63]. Only a single study included in this MA evaluated VE derivatives beyond AT with 180 mg TT + 40 mg AT but this intervention failed to significantly decrease MDA levels [42]. Future studies to evaluate the potential benefits of different VE derivatives would prove beneficial.

On average, more than 90% of American adults fail to meet the RDA for VE, with significantly greater implications for HD patients given the higher levels of malnutrition and OS [64]. The US RDA is 14 mg (approximately 18 IU/day) [64], yet most American VE supplementation occurs at high dosage ≥400 IU/day [65]. Supplementation in deficient individuals is more likely to be beneficial, however, VE intake ≥400 IU/day has been associated with increased all-cause mortality [47]. Variation in VE dietary intake and supplementation may explain the pro-oxidant effects observed in high dose VE studies. Conversely, it was found that 400 IU of AT was the minimum dose required to show a positive effect on ox-LDL [66]. VE significantly decreased ox-LDL levels in hypercholesterolaemic smokers compared to individuals that were smokers but non-hypercholesterolaemic or vice versa [67]. This suggests the beneficial antioxidant effects associated with VE may be limited to only those with elevated levels of OS. VE catabolites (carboxyethyl hydroxychromans (CEHC)) are present at higher plasma levels among patients with deteriorating renal function [68]. Alpha and gamma CEHC have shown strong antioxidative properties and the accumulation of these catabolites is thought to be responsible for the increased potency of VE in haemodialysis patients. As a result, higher-dosage VE supplementation in HD patients may be safer and more efficacious in comparison to healthy individuals although addressing the challenges of individual tailored VE levels in HD patients may prove difficult [68].

VE IM administration showed more than double the effect size compared to oral delivery, which may be due to variable metabolic effects on MDA levels [69]. In addition, IM multivitamin supplementation has been reported to improve plasma vitamin levels compared to oral administration [70]. Subgroup analysis from 11 trials failed to detect any differences between serum/plasma and non-serum/plasma-based biomarkers. Previous concerns of volumetric changes in patients during HD affecting MDA measurements [71] led to proposed alternative standardised measures based on plasma/serum MDA to cholesterol ratios [37].

Four studies included in this MA were RCTs [39, 41–43], while the remainder were open-labelled/non-RCTs. Strong correlations between RCTs and non-RCTs were previously demonstrated, although non-RCTs demonstrate both greater treatment effects and heterogeneity, in line with our findings [72]. Larger double-blind placebo controlled RCTs with HD patient controls are necessary to confirm these findings. Only two studies considered duplicate MDA measurements before VE supplementation for which an average value provided a more robust measure of MDA over time, given its intrinsic variability [31, 32].

Nine studies were conducted pre 2000 and best practice normally suggests study quality improves with time. As lower treatment effect estimates tend to correlate with higher study quality, less extreme results are more likely in recent studies [73]. The effectiveness of the trials reported between 1984 and 2016 appears to have diminished (Fig. 2), a phenomenon commonly referred to as “fading of reported effectiveness” [74].

While MDA remains a commonly used biomarker to quantify OS, concerns remain over its sensitivity, especially with the thiobarbituric acid reactive substances (TBARS) assay, the most popular MDA measurement method [75]. These concerns relate to the reproducibility of the TBARS assay and the production of other lipid peroxidation by-products [76]. HPLC and gas chromatography mass-spectrometric (GC-MS) assays are now considered more sensitive and reproducible [77]. Fourteen of the trials in our MA used the TBARS assay, with only a single trial using HPLC showing a non-significant decrease in MDA [37], although this 24-h trial accounted for only a single HD and VE supplementation period. This is an area for future consideration as HPLC/GC-MS data becomes more widely available. A high level of reactivity and instability have also over-shadowed the reliability of MDA [78], given its cross-reactivity and rapid enzymatic degradation by mitochondrial aldehyde dehydrogenase [79]. This highlights the necessity for an improved OS biomarker, which would address a wide range of markers to provide a more robust measure of TAC. A 2011 review analysing prospective studies found OS biomarkers were not predictive of CVD, questioning the sensitivity of OS as a clinical measurement [80]. While the use of HPLC following TBARs can improve the specificity of MDA quantification, the spectrum of MDA measurement approaches makes comparisons between measurement modalities challenging. This, coupled with concerns regarding the specificity and sensitivity of the TBARS assay to accurately quantify MDA, questions its reliability as an absolute indicator of lipid peroxidation, as opposed to a broad predictor of OS [81].

Our study had several strengths. Meta-analyses provide robust estimates of the true effect size affected by the limitations of smaller individual studies. The comprehensive and transparent search strategy used reduced the possibility of excluding relevant studies and assessed levels of heterogeneity between studies. Our study also had several limitations. There was potential publication bias toward smaller studies with less significant findings, given the asymmetry observed in the funnel plot (Fig. 3). The significant I^2^ value of 90% highlighted the heterogeneity and inconsistency in the study design and treatment effect across trials, which is unsurprising given the variation observed in VE supplementation, therapy duration, and MDA measurement methods. The high level of heterogeneity raises concerns on the validity of the summary estimate and may reduce the robustness of our conclusions.

The relationship between VE and OS in HD patients has been well documented, but the optimal levels to reduce OS remains unclear, largely due to the pro-oxidant effects of VE at higher dosage and a lack of a defining threshold. The requirements of increased OS in HD patients may not be representative of similar challenges in the general population. Evaluation of VE effects has largely focused on AT while other forms of VE may show enhanced antioxidant properties especially when co-supplemented, given their unique properties. Supplementation of VE in isolation ignores the potential synergistic benefits provided by other compounds and excess AT may result in antioxidant imbalance. The accuracy for the quantification of OS and TAC remains challenging with wide-ranging variation in the biomarkers used to measure OS in response to supplementation.

## Conclusions

Our study supports the use of VE supplementation in reducing OS in HD patients although concerns over the validity of our findings remain given the high levels of heterogeneity observed between studies. Improved measurement and reporting of future MDA assays will elucidate the true potential of VE supplementation on OS in HD patients to reduce morbidity and mortality and improved quality of life for HD patients [82].

## Data Availability

The datasets used and/or analysed during the current study are available from the Principal Investigator (Gareth McKay: g.j.mckay@qub.ac.uk) upon request.
